# Comparison of DNA preservation and ATR-FTIR spectroscopy indices of cortical and trabecular bone of metacarpals and metatarsals

**DOI:** 10.1038/s41598-023-41259-2

**Published:** 2023-09-19

**Authors:** Tamara Leskovar, Jezerka Inkret, Irena Zupanič Pajnič, Ivan Jerman

**Affiliations:** 1 Centre for Interdisciplinary Research in Archaeology, Department of Archaeology, Faculty of Arts, University of Ljubljana, Zavetiška 5, 1000 Ljubljana, Slovenia; 2 Institute of Forensic Medicine, Faculty of Medicine, University of Ljubljana, Korytkova 2, 1000 Ljubljana, Slovenia; 3 National Institute of Chemistry, Hajdrihova 19, 1000 Ljubljana, Slovenia

**Keywords:** DNA, Skeleton

## Abstract

Shape, size, composition, and function of the bones in the human body vary on the macro, micro and nanoscale. This can influence changes caused by taphonomy and post-mortem preservation, including DNA. Highly mineralised compact bone is less susceptible to taphonomic factors than porous trabecular bone. Some studies imply that DNA can be better preserved in trabecular bone, due to remnants of the soft tissue or bacteria better digesting organic matter while not digesting DNA. The aim of this study was to understand the differences between compact (diaphyses) and trabecular (epiphyses) bone on a molecular level and thus the reasons for the better preservation of the DNA in the trabecular bone. The powder obtained from epiphyses and diaphyses of metacarpals and metatarsals was analysed using ATR-FTIR spectroscopy and compared. Samples with poorest DNA preservation originated from diaphyses, predominantly of metatarsals. They were characterised by higher concentrations of phosphates and crystallinity, while lower collagen quality in comparison to samples with the best DNA preservation. Epiphyses presented higher concentrations of better-preserved collagen while diaphyses had higher concentrations of carbonates and phosphates and higher crystallinity. Due to better-preserved collagen in the epiphyses, the soft tissue remnants hypothesis seems more likely than the bacteria hypothesis.

## Introduction

The structure of bone is very heterogeneous, consisting of cells, an inorganic matrix, and an organic matrix. The inorganic part of the bone is formed by the hydroxyapatite mineral analogue, with various substitutions and vacancies in the crystal matrix. The organic part is formed by collagen and non-collagenous proteins. In humans, two types of bone can be found, trabecular and cortical. The first is a porous, flexible, reinforcing tissue that contains bone marrow while the latter is less porous, stronger, and denser^1^. As different bones and their parts perform different functions in the body, there is also some inter-and intra-bone variability in their shape, size, and composition on the macro, micro and nanoscale^2,3^.

Variation in the bone composition can highly influence the changes in the bones caused by taphonomy and thus post-mortem preservation of the bone, including on the molecular level. The general view is that highly mineralised compact bone, especially of the diaphysis, is less susceptible to taphonomic factors than more porous trabecular bone^4,5^. The same idea is commonly applied to the preservation of DNA in the bones. However, recent studies on contemporary and Second World War skeletons imply that small skeletal elements, especially small bones of the hands and feet are the richest source of DNA^6–8^. Furthermore, studies also report intra-bone variability in the DNA yield^6^, including metacarpals and metatarsals^9^.

Metacarpals and metatarsals are composed of a shaft—diaphysis and a proximal and a distal end—epiphyses. The diaphysis is predominantly formed by dense compact bone, while the epiphyses are by porous trabecular bone, covered with a thin layer of cortical bone^10^. As the dense compact bone is supposedly less susceptible to taphonomy than more porous trabecular bone^4,5^, higher DNA yield would be expected in the former. However, the study of Inkret et al.^9^ showed that epiphyses are a richer source of DNA than diaphysis, especially the epiphyses of metacarpals. Based on these unexpected results two hypotheses were proposed. The first hypothesis states that the epiphyses have lower inorganic material content and higher carbon content from the soft tissue. Remnants of soft tissue are stored in the intertrabecular spaces^11^, where they are protected against taphonomic processes and contribute to a higher DNA yield^12^. The second hypothesis states that certain bacterial taxa may be better adapted to degrading organic matter of bone and do not digest DNA directly^13^. Bacterial infestations can thus aid skeletal integrity and contribute to a higher DNA yield in trabecular bone^14^.

The main objective of this study was to apply Attenuated total reflectance (ATR) Fourier Transform Infrared (FTIR) spectroscopy to better understand the differences between compact (diaphyses) and trabecular (epiphyses) bone on a molecular level and thus the reasons for the better preservation of the DNA in the trabecular bone reported by Inkret et al.^9^. To avoid repetition, only results on the DNA analyses are included here (Supplementary material Table 1), while other details on DNA part of the research can be found in the cited article by Inkret et al.^9^. Due to ease of use and relatively low cost, ATR-FTIR spectroscopy is an increasingly used technique in various fields. It is commonly applied to the study of human skeletal remains, including DNA preservation in the bone^15–23^. Using only a small amount of a sample, the technique provides fast, yet reliable results on the molecular composition of the studied material. Exploring the obtained spectra, be it directly or with the introduction of FTIR indices, it is possible to explore the nature of the molecular bonds, their environment, and their relative content^24^. Applied to skeletal tissues, ATR-FTIR spectroscopy provides information on the organic and inorganic components in the bone and enables the detection of small chemical and/or structural variation in the samples^15,25,26^. As chemico-physical properties of skeletal tissues are highly heterogeneous, characterisation of their molecular structure using FTIR spectra directly can be elusive^27^. Consequently, straightforward investigation of spectral bands is usually replaced by the calculations of the ratios between peak heights or/and areas, investigating relative and not absolute concentrations of bone components^28^.

Recent studies^6,7^ show that the preservation of DNA in the skeletonised remains differs among types of skeletal elements. Though these studies clearly indicate that small skeletal elements with high content of trabecular bone are a rich source of DNA, the reasons for this are not yet clarified. Trying to understand why DNA was better preserved in the epiphyses and not diaphyses of the metacarpals and metatarsals, the powder obtained from epiphyses and diaphyses was analysed using ATR-FTIR spectroscopy and compared. All together 193 metatarsals (MT) and metacarpals (MC) were collected from a singular Second World War mass grave (Konfin Shaft 2, Loški potok, Slovenia), ensuring that all the samples were exposed to the same gross environmental conditions in the same geographical location for the same amount of time.

## Results

In the FTIR spectra and indices of analysed samples the main building blocks of bones, carbonated hydroxyapatite and collagen are easily recognised. Collagen is best seen in peaks at 1240 cm^−1^ (Amide III), 1545 cm^−1^ (Amide II) and 1640 cm^-1^ (Amide I), phosphates in peaks at 500 cm^−1^, 600 cm^−1^, 960 cm^−1^ and 1010 cm^−1^, and carbonates in peak at 872 cm^−1^. Also present are peaks at 1410 cm^−1^ and 1440 cm^−1^, corresponding to carbonates with collagen sideband. The taphonomically induced changes in the bones were observed through the FTIR indices R960/1115*, which is correlated to the crystallinity, and R1240/1445 and H1330, which reflect collagen denaturation.

### DNA clusters

Based on the Auto and Auto/Deg highest Silhouette scores (0.827) were obtained for three clusters (C), so samples were separated into three clusters (Table 1). Cluster 1 (C1) presented highest DNA quality (Auto/Deg ratio), cluster 2 (C2) highest DNA quantity (Auto) and cluster 3 (C3) lowest DNA quantity and quality.

**Table 1:** Clusters based on autosomal and degradation targets.

Cluster	Auto	Auto/Deg ratio
C1	0.0600 ± 0.077	10.55 ± 13.10
C2	0.681 ± 0.437	28.44 ± 19.92
C3	0.015 ± 0.049	310.82 ± 17.11

### Spectra

**Figure 1:**
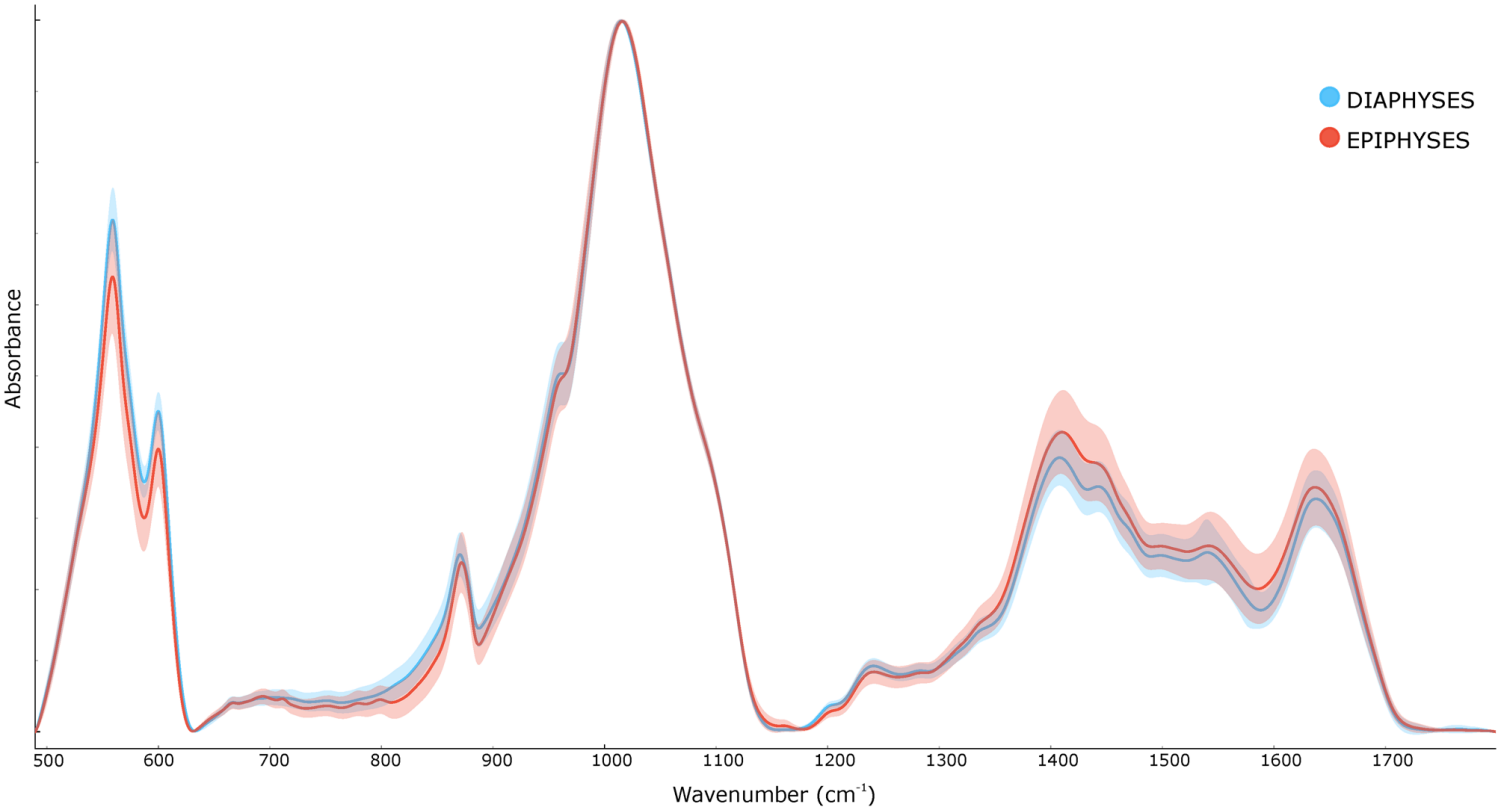
Averaged spectra of the diaphyses (blue) and epiphyses (red) combined for MC and MT.

Seven outliers were detected and excluded (samples 215, 249, 254, 419, 496, 498 and 682) from further analyses. Samples were excluded due to an unusually high peak at 720 cm^−1^ indicating carbonate contamination, and several unusually high peaks in the domain between 1400 and 1600 cm^−1^ (Supplemental files, Fig. 1).

Visual observation of the averaged spectra of the epiphyses and diaphyses presented some obvious differences in the heights of the phosphate peaks at 560 cm^−1^, 600 cm^−1^, carbonate with collagen side band peaks at 1410 cm^−1^, 1445 cm^−1^, collagen peak at 1640 cm^−1^,and some less obvious but detectable differences in the 872 cm^−1^ carbonate peak,1240 cm^−1^ Amide III peak and 1545 cm^−1^ Amide II peak. On average, peaks corresponding to the mineral components in the bone (phosphates and carbonates) are higher in the diaphyses, while peaks corresponding to the organic components (collagen) in the bone are higher in the epiphyses (Fig. 1).

**Figure 2:**
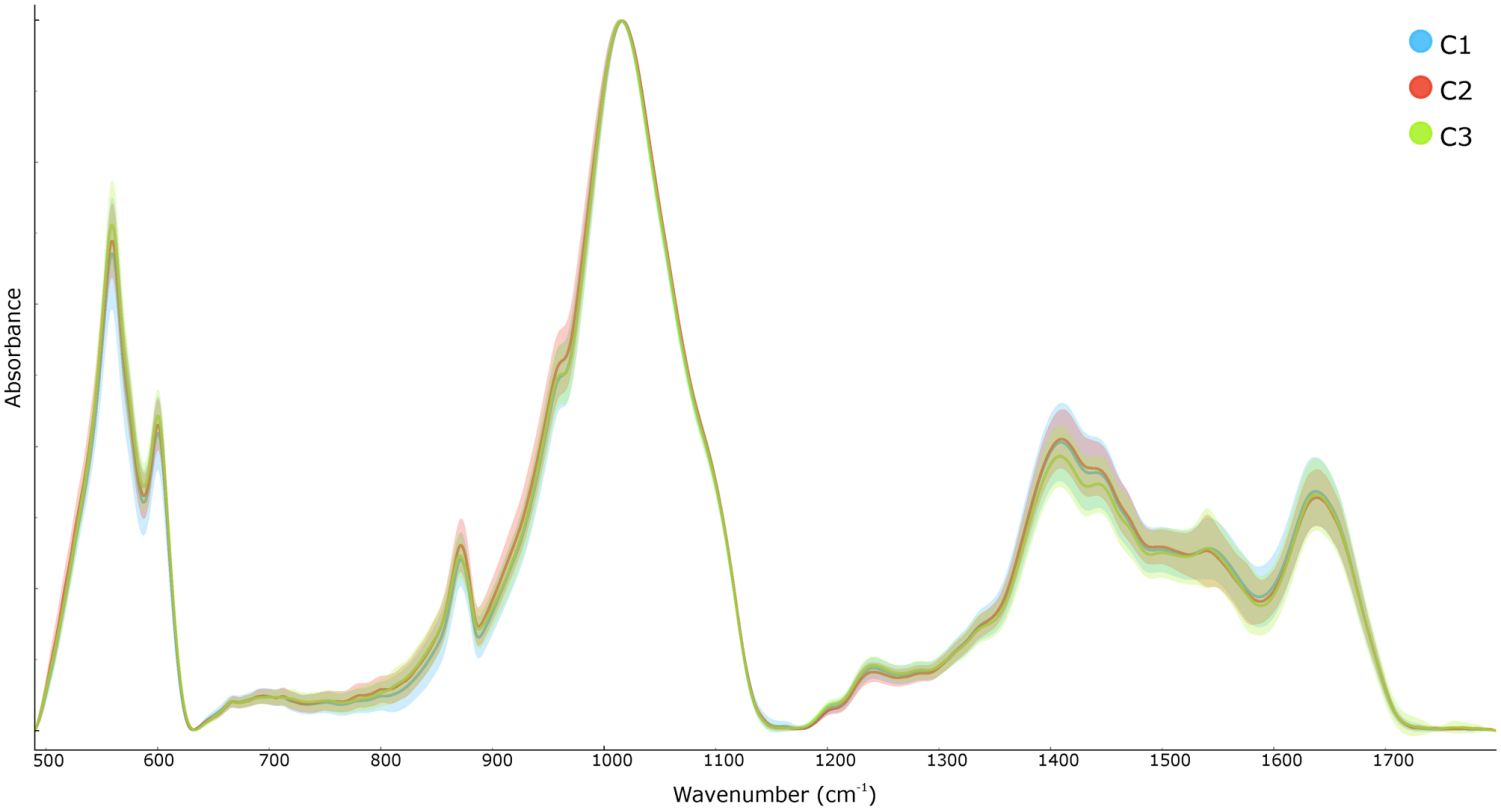
Averaged spectra of the samples in the C1 (blue), C2 (red) and C3 (green).

Visual observation of averaged spectra of samples from C1, C2 and C3 presented a variety of small differences. Samples from C3 have the highest phosphate peaks at 560 cm^−1^ and 600 cm^−1^, samples from C2 have the highest carbonate peak at 872 cm^−1^, and carbonate with a collagen sideband peak at 1410 cm^−1^, while samples from C1 have lowest phosphate peaks at 560 cm^−1^, 600 cm^−1^, and carbonate peak at 872 cm^−1^, while highest Amide I peak at 1640 cm^−1^ (Fig. 2).

### FTIR indices

According to Mann–Whitney U Test, significant differences (p ≤ 0.05) between diaphyses and epiphyses are present in all the FTIR indices. Epiphyses have lower R1245/1445, H600, H560, H1240, H1330, H872 and R960/1115* in comparison to diaphyses (Table 2). Epiphyses present lower relative concentrations of phosphates, carbonates and Amide III, lower crystallinity, and lower collagen denaturation when compared to diaphyses.

**Table 2:** Results on chemometric indices of epiphyses and diaphyses (averages with ranges).

FTIR index	Epiphyses	±	Diaphyses	±	p
R1245/1445	0.2238	0.0412	**0.2689**	0.0171	0.000
H600	0.3978	0.0526	**0.4509**	0.0262	0.000
H560	0.6405	0.0797	**0.7205**	0.0442	0.000
H1240	0.0842	0.0165	**0.0927**	0.0107	0.000
H1640	**0.3454**	0.0538	0.3289	0.0406	0.001
H1330*	0.00019	0.00008	**0.000229**	0.00011	0.001
H872	0.2391	0.0428	**0.2496**	0.0302	0.006
R960/1115*	2.68	0.93	**2.93**	0.96	0.011
H1540	**0.2630**	0.0476	0.2527	0.0466	0.032

*FTIR indices are ordered by relevance, highest peaks and ratios are in bold.

Significant differences (p < 0.05) between DNA clusters C1, C2 and C3 are present in R1240/1445, H560, H600, and R960/1115*. Samples in C1 have the lowest H560 and H600, reflecting lowest relative concentrations of phosphates. Samples in C2 have the lowest collagen denaturation (R1240/1445) and crystallinity (R960/1115*). Samples in C3 have the highest collagen denaturation (R1240/1445), highest relative concentrations of phosphates (H560, H600) highest relative concentrations of Amide III (H1240), and highest crystallinity (R960/1115*) Table 3).

**Table 3:** Results on FTIR indices of samples from the DNA clusters (averages with ranges).

FTIR index	C1	±	C2	±	C3	±	p
R1240/1445	0.2439	0.0396	0.2257	0.040	0.2654	0.0244	0.000
H560	0.6723	0.0779	0.6899	0.0519	0.7128	0.0613	0.000
H600	0.4196	0.0514	0.4309	0.0356	0.4431	0.0366	0.002
H1240	0.0881	0.0148	0.0829	0.0138	0.0921	0.0125	0.027
R960/1115*	2.796	0.969	2.378	0.774	2.971	0.905	0.05

## Discussion

Samples with the poorest overall DNA preservation in cluster C3 with the lowest DNA quantity (Auto) and poorest DNA quality (Auto/Deg ratio) have highest crystallinity, highest relative concentrations of phosphates, and highest collagen denaturation. Different studies report different strength of correlation between DNA preservation and the relative concentrations of collagen and carbonates, crystallinity, and collagen quality in the bone^18,29–34^. The reason for such differences could be the state and/or origin of the samples included. Research shows that differences in the FTIR indices of bone are caused by numerous reasons, such as the taphonomic history of the remains, postmortem interval, and inter and intra skeletal variation^15,22,29,34–36^. Here presented results show that when combining epiphyses (predominantly trabecular bone) and diaphyses (predominantly cortical bone) of metacarpals and metatarsals, better DNA preservation is indicated by lower collagen denaturation, lower relative concentrations of phosphates and lower crystallinity.

Despite being significant, the differences between samples with different DNA preservation are small. A previous study dealing with minor differences in the samples used to assess DNA preservation^34^ already showed that ATR-FTIR spectroscopy has its limitations. These limitations are even more obvious here, as the samples originated from the remains exposed to the same gross environmental conditions in the same geographical location for the same amount of time. Also, sampling was limited to metacarpals and metatarsals, with the only major difference in the part of bone (epiphyses vs. diaphyses). Furthermore, metacarpals and metatarsals present a good balance between all the major components in bone, leading to a mutual protection between minerals and collagen^37,38^. This could explain why significant differences were relatively limited. The most tangible difference was collagen denaturation, clearly more advanced in the samples with the poorest DNA quality and lowest DNA quantity, which is in an agreement with previous studies^18,30,33,39^. Higher crystallinity and relative concentrations of phosphates in the samples with the poorest DNA quality and lowest DNA quantity could be a consequence of the observed significant losses of collagen and consequent faster recrystallization process of the mineral as collagen and mineral mutually protect each other^38,40^.

According to the visual observation, peaks corresponding to the mineral components in the bone (phosphates and carbonates) were higher in the diaphyses, while peaks corresponding to the organic components (collagen) in the bone were higher in the epiphyses (Fig. 1). A more tangible and objective comparison of FTIR indices showed that when compared to diaphyses, epiphyses have significantly higher peaks correlated to collagen (peaks at 1540 cm^−1^ and 1640 cm^−1^). The outlier is the peak of Amide III (at 1240 cm^-1^), higher in the diaphyses. The reason for higher Amide III peak in diaphyses needs further investigation. It could be due to its weak signal^41^ or the fact, that contrary to Amide I, Amide III is not affected by water absorption^42^. Additionally, collagen denaturation (R1240/1445 and H1330), crystallinity (R960/1115*), and relative concentrations of carbonates (peak at 872 cm^−1^) are lower in the epiphyses. This agrees with higher 560 cm^−1^ and 600 cm^−1^ peaks in the diaphyses, as the peaks mainly correspond to phosphates in the bone.

Based on the results it seems like epiphyses have higher concentrations of better-preserved collagen and diaphyses have higher concentrations of carbonates and phosphates and higher crystallinity. This is in accordance with the bone structure of cortical (diaphyses) and trabecular (epiphyses) bone^10^. The first has a higher percentage of inorganic material while the second has a higher content of organic material as the soft tissue is stored in the intertrabecular spaces^11^. Regarding the two stated hypotheses of why a higher DNA was found in epiphyses and not diaphyses in the study of Inkret et al.^9^, the first one seems more likely. The higher concentrations of organic components in trabecular bone could be a consequence of the soft tissue remnants. On the other hand, the second hypothesis of an increased presence of bacteria adapted to degrading organic matter of bone in trabecular bone^13,43^ would likely cause more damage to the organic components of trabecular than cortical bone. As collagen is better preserved in trabecular bone, the second hypothesis seems less likely.

## Conclusions

The study of Inkret et al.^9^ showed that epiphyses (more trabecular than compact bone) are a richer source of DNA than diaphysis (more compact than trabecular bone). It was hypothesised that the reason for better DNA preservation in the epiphyses is either a lower inorganic material content and higher carbon content or that certain bacterial taxa may be better adapted to degrading organic matter of bone and do not digest DNA directly. The main objective of this study was to better understand the differences between compact (diaphyses) and trabecular (epiphyses) bone on a molecular level and thus the reasons for the surprisingly better preservation of the DNA in the latter. Since all the samples originated from the metacarpals and metatarsals of the skeletal remains with the same taphonomic history, the environmental impact on the preservation state of the samples is minimal. Thus, the differences in the state of organic and inorganic components of the samples and DNA preservation can mainly be attributed to the differences in the type of bone (trabecular vs. compact).

Samples with the poorest DNA preservation mainly originated from diaphyses and were characterised by higher relative concentrations of phosphates and crystallinity, while lower collagen quality in comparison to samples with the best DNA preservation. However, the differences were small, which could partially be explained by the similar gross characteristics of the samples (skeletal element, taphonomic history). Significant differences in FTIR indices were observed between epiphyses and diaphyses. The former presented higher concentrations of better-preserved collagen while the latter had higher concentrations of carbonates and phosphates and higher crystallinity. Due to better-preserved collagen in the epiphyses, the soft tissue remnants hypothesis seems more likely than the bacteria hypothesis.

A drawback of dealing with the remains from the Second World War is their prolonged (almost 80 years) exposure to taphonomic factors, which caused DNA degradation. Consequently, the preserved quantity of DNA was too low to produce tangible results from ATR-FTIR analyses on the isolates directly.

## Material and methods

### Samples

Right metacarpals and metatarsals were sampled from the remains excavated from the mass grave Konfin II. Mass grave was discovered in 2007 and excavated according to the Slovenian Act on War Graves (Article 27 and 28)^44,45^. The excavation was performed by the Institute of Forensic Medicine, Faculty of Medicine, University of Ljubljana, Slovenia. The same institute performed DNA analyses and stores the samples, which were also available for FTIR analyses, performed at the National Institute of Chemistry in Ljubljana, Slovenia. 382 samples were processed, 193 diaphyses and 189 epiphyses (see Supplemental files, Table 1). A rotatory sanding tool was used to mechanically clean all the bones and remove surface contamination. Bones were then chemically cleaned using 5% Alconox detergent and 80% ethanol and left overnight to dry. Using a sterilized diamond saw, each bone was cut to separate proximal and distal epiphyses and diaphysis. Both epiphyses were combined in one sample.

The diaphysis and combined epiphyses from each MT and MC bone were separately ground into a fine powder using a Bead Beater MillMix 20 homogenizer. Powdering was performed for 1 min at a frequency of 30 Hz in metal grinding vials with metal balls. Before powdering, the metal vials and bone fragments were cooled with liquid nitrogen to prevent overheating. Cleaning and powdering were performed in a room designed exclusively for processing old skeletal remains. All working surfaces and all the equipment used were cleaned with bleach, water, and ethanol, and were exposed to UV irradiation overnight.

The DNA quantity (Auto target) and DNA degradation (Auto/Deg ratio or degradation index) were determined with the real time PCR (qPCR) method using the PowerQuant System (Promega)^9^, and used as DNA indexes.

### ATR-FTIR spectroscopy

FTIR spectroscopic analyses were performed using a Bruker Vertex 70 equipped with a diamond ATR (Attenuated Total Reflection) accessory and MCT (Mercuric Cadmium Telluride) detector. All the samples were scanned in the domain between 400 and 4000 cm^-1^. The spectrum of each sample was collected from an average of 64 scans at a resolution of 1 cm^-1^. The baseline was subtracted using Iterating Averaging^46^, and each spectrum was normalized to the highest peak (phosphate peak = *v*_*3*_PO_4_ at ~ 1010 cm^−1^).

To overcome the overlapping of some of the peaks, Savitzky–Golay second derivative with five points of window was performed on the domain between 500 and 1800 cm^-1^.

Obtained spectra and FTIR indices were both included in the analyses. FTIR indices were extrapolated from the normalized spectra and second derivative (marked with *) spectra (Table 4). To explore as many differences in the organic (collagen represented by amides) and mineral (apatite presented by phosphates and carbonates) components of the bone as possible, various FTIR indices cited in the previous studies using FTIR spectroscopy to investigate skeletal remains were incorporated.

**Table 4:** FTIR indices extracted from the spectra.

Index	Peaks (cm^−1^)	Meaning	Reference
R960/1115*	Ratio between 960 and 1115	Crystallinity index I (stoichiometric vs. non-stoichiometric apatite)	^26^
*H560*	Height of 560	Non-stoichiometric apatite	^47–49^
*H600*	Height of 600	Stoichiometric apatite	^47–49^
*H872*	Height of 872	Relative concentration of A + B-type carbonates	^50,51^
*H1240*	Height of 1240	Relative concentration of amide III	^24^
H1330*	Height of 1337	Collagen denaturation	^52^
*H1640*	Height of 1640	Relative concentration of amide I	^53,54^
*H1545*	Height of 1545	Relative concentration of amide II	^24^
*R1240/1445*	Ratio between 1240 and 1445	Collagen denaturation	^55^

### Statistical analyses

Statistical analyses were performed using Orange^56^ and IBM SPSS 26. Outliers were first detected and excluded by analyzing baseline corrected normalized spectra with detection method. Local Outlier Factor (Contamination: 0.02; Neighbors: 20; Metric: Euclidian). All the following analyses were performed on the remaining samples. K-means clustering algorithm (Number of Clusters: From 2 to 8; Preprocessing: Normalize columns; Initialization: KMeans +  + ; Re-runs: 10; Maximum iterations: 300) was used to separate the samples into clusters based on DNA quantity (autosomal target = Auto) and DNA quality (Auto/Deg ratio) (see Ref.^9^ and Supplementary material Table 1 for details). Silhouette scores, which contrast the average distance to elements in the same cluster with the average distance to elements in other clusters (the higher the score, the better the clustering) were used to separate the samples in the best number of clusters. Comparisons in the spectra and FTIR indices were performed between the DNA clusters and between the epiphyses and diaphyses.

Shapiro–Wilk test was used to explore the normality of the data. Based on the results, comparisons were made using the nonparametric Independent Samples Mann–Whitney U test.

Significance was defined with p ≤ 0.05.

### Ethics approval

The research was performed in accordance with the Declaration of Helsinki and was approved by the Slovenian Medical Ethics Committee (0120-481/2018-11 and 0120-350/2018/6). The individuals included in the elimination database gave their written informed consent.

### Supplementary Information

Supplementary Figure 1.

Supplementary Table 1.

Supplementary Table 2.

## Data Availability

The datasets generated during and/or analysed during the current study are available from the corresponding author on reasonable request.
