# Supplementary material for: Comparison of DNA preservation and ATR-FTIR spectroscopy indices of cortical and trabecular bone of metacarpals and metatarsals
Source: Sci Rep. 2023 Sep 19;13:15498. doi: 10.1038/s41598-023-41259-2 (PMC10509243; doi:10.1038/s41598-023-41259-2)
Supplement: Supplementary file 1 — Supplementary Figure 1. [file 41598_2023_41259_MOESM1_ESM.pdf]

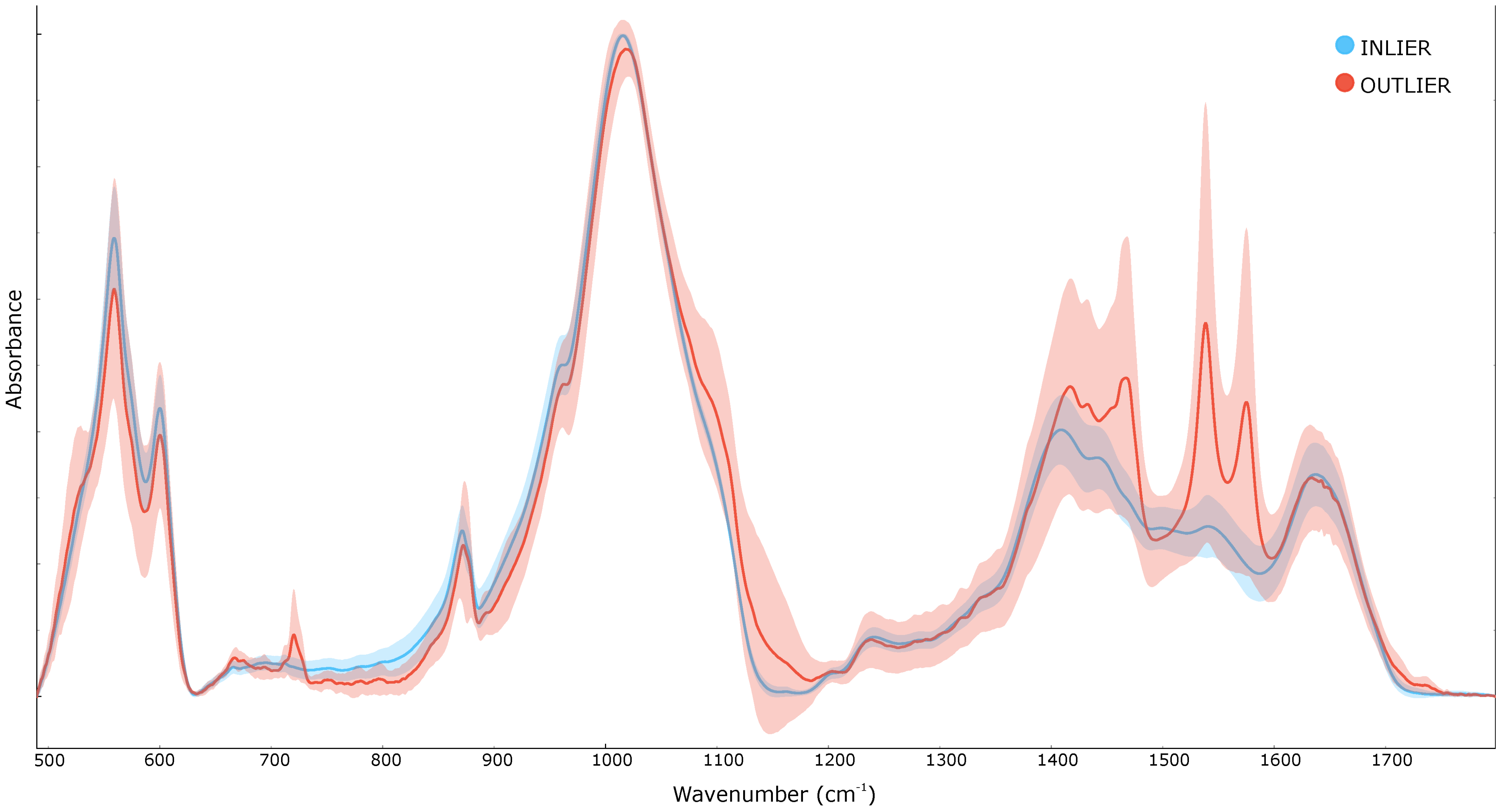

SM Figure 1: Seven outliers with unusually high peak at 720 cm^{-1} and several unusually high peaks in the domain between 1400 and 1600 cm^{-1}.
